# Decrease of FSTL1-BMP4-Smad signaling predicts poor prognosis in lung adenocarcinoma but not in squamous cell carcinoma

**DOI:** 10.1038/s41598-017-10366-2

**Published:** 2017-08-29

**Authors:** Jean Chiou, Chia-Yi Su, Yi-Hua Jan, Chih-Jen Yang, Ming-Shyan Huang, Yung-Luen Yu, Michael Hsiao

**Affiliations:** 1 The PhD. Program for Cancer Biology and Drug Discovery, China Medical University, Taichung, Taiwan; 2 Genomics Research Center, Academia Sinica, Taipei, Taiwan; 3 Graduate Institute of Bioengineering, University of Washington, Seattle, WA USA; 4 Department of Internal Medicine, Kaohsiung Medical University Hospital, Kaohsiung Medical University, Kaohsiung, Taiwan; 5 Graduate Institute of Biomedical Sciences and Center for Molecular Medicine, China Medical University, Taichung, Taiwan; 6 Department of Biochemistry, College of Medicine, Kaohsiung Medical University, Kaohsiung, Taiwan

## Abstract

Follistatin-related protein 1 (FSTL1) plays a critical role in lung development through regulating BMP4-p-Smad1/5/8-Smad4 pathway. Regarding that many developmental pathways in embryogenesis are dysregulated in cancer, we aim to unravel the role of FSTL1-BMP4-Smad pathway in lung cancer. Our results showed low FSTL1 immunoexpression was significantly correlated with poor prognosis while patients with low BMP4 or low Smad4 immunoexpression showed a trend toward poor prognosis. When stratified by different histological types, low FSTL1, BMP4, and Smad4 expression retained their trends in predicting poor prognosis in lung adenocarcinoma (LUAD) but not in lung squamous cell carcinoma (SCC). Low FSTL1, BMP4, and Smad4 expression were more frequently observed in LUAD patients with smoking history. To determine smoking effect on FSTL1, normal cell BEAS2B and lung cancer cell lines was treated with nicotine and the results showed nicotine increased the proliferation of these cells. Interestingly, FSTL1 attenuated nicotine-induced BEAS2B and lung cancer cell line proliferation. Altogether, low FSTL1, BMP4, and Smad4 expression significantly correlated with poor prognosis in LUAD but not in SCC. Frequent decrease of FSTL1 expression in smokers LUAD further indicates its importance and therapeutic potential for lung cancer patients with specific subtypes. FSTL1 may prevent nicotine-induced lung cancer cell proliferation.

## Introduction

Lung cancer as one of the most common cancer type worldwide has a high mortality rate in spite of the improvement of therapeutic strategies^1^. Diverse histological and molecular subtypes make lung cancer a complicated disease and make further investigation a more difficult task^2^. For adenocarcinoma, the dominant histological subtype of lung cancer, increased understanding of molecular pathogenesis such as EGFR mutation, KRAS mutation and ALK fusion leads to the success of targeted therapy^3^. By contrast, squamous cell carcinoma tend to have different mechanisms of tumorigenesis and progression. Targeted therapies used in adenocarcinoma are mostly ineffective for patients with squamous cell carcinoma^4, 5^. These valuable experiences give us a lesson that discovering novel targets specific for certain histologic or molecular subtypes lead to the greatest therapeutic benefit^6^.

Bone morphogenetic proteins (BMPs), belonging to the transforming growth factor â (TGF-â) superfamily, have received a tremendous wave of interest by cancer researchers for their multi-faceted functions in regulating critical cellular processes such as apoptosis and differentiation during embryonic development^7–9^. Bone morphogenetic proteins 4 (BMP4), a member of BMP family^10^, involves in many aspects of cancer progression including cancer cell growth^11^, differentiation^12^, apoptosis^13^, and migration and invasion^14, 15^ and has different effects on different cancer types^16^. In lung cancer, premature senescence induced by BMP4 mediated Smad signaling pathway decreases cell growth rate *in vitro* and tumorigenicity *in vivo* ^17–19^. Inhibition of invasion ability and less angiogenic phenotype were also seen in lung cancer cells with BMP4 treatment^20^.

Follistatin-related protein 1 (FSTL1), primarily derived as a TGF-β inducible protein^21^, is reported to be decreased and undetectable in various human cancer cells but was detected in various mouse organs with the highest expression in lung tissue^22^. Further research show that FSTL1 expression inhibits cell growth and participates in suppression of invasion and metastasis in lung cancer cells^23, 24^. Recently, FSTL1 is also recognized as a critical developmental regulator for organogenesis especially in lung development^25–28^. *Fstl1*-deficient mice have impaired alveolar maturation due to dysfunction in distal alveolar differentiation and insufficient surfactant production^28, 29^. FSTL1 is shown to interfere in BMP4-p-Smad1/5/8-Smad4 pathway mediated surfactant expression^29^. Since many developmental pathways in embryogenesis play critical roles in oncogenesis and cancer progression^30, 31^, the importance of FSTL1-BMP4-Smad pathway in lung development suggests its role in cancer is worthy of investigation. However, the value of FSTL1-BMP4-Smad pathway in clinical lung cancer patients is still largely unknown.

In this study, we showed the prognostic significance of FSTL1-BMP4-p-Smad1/5/8-Smad4 pathway in lung cancer with histological specificity. Lung adenocarcinoma patients with low FSTL1, BMP4, or Smad4 expression had poor prognosis. In contrast, markers in FSTL1-BMP4-p-Smad1/5/8-Smad4 pathway have no prognostic value in lung squamous cell carcinoma patients. Moreover, frequent decrease of FSTL1-BMP4-p-Smad1/5/8-Smad4 pathway observed in smokers and in ALK-fusion or KRAS mutant adenocarcinoma patients may facilitate the understanding of the molecular pathogenesis of lung adenocarcinoma. We also presented *in vitro* and *in vivo* experiments to decipher the interplay between nicotine and FSTL1.

## Materials and Methods

### Patient case selection and ethics statement

A total of 96 patients diagnosed with non-small cell lung cancer including 58 cases of adenocarcinoma, 32 cases of squamous cell carcinoma, and 6 cases of large cell carcinoma at the Kaohsiung medical university Hospital of Taiwan from 1991 to 2007 were included in this study. All patients received standard treatment protocols according to hospital guidelines. Patients with operable stage I-III NSCLC underwent lobectomy or pneumonectomy with mediastinal lymphadenectomy. No adjuvant chemotherapy was conducted for patients with completely resected stage I NSCLC. Patients with resectable stage II and III NSCLC were treated with postoperative adjuvant platinum-based chemotherapy. Patients with unresectable locally advanced or metastatic disease received chemotherapy with or without radiotherapy. Follow-up data were available in all cases, and the longest clinical follow-up time was 190 months. All cases were staged according to the cancer staging manual of the American Joint Committee on Cancer and the histological cancer type was classified according to World Health Organization classification.

This study was approved by the ethics committees of Institutional Review Board of Kaohsiung Medical University Chung-Ho Memorial Hospital (KMUH-IRB-E(I)-20160099) and was carried out in accordance with the approved guidelines. No informed consent was required because the data were analyzed anonymously and no identifying information relating to participants were included.

### Tissue microarray construction and immunohistochemistry staining

Representative three 1-mm-diameter cores from each tumor taken from the formalin-fixed paraffin embedded tissues were selected by morphology typical of the diagnosis. Immunohistochemical (IHC) staining was performed on serial 5-micrometer-thick tissue sections cut from the tissue microarray (TMA). IHC staining of FSTL1, BMP4, and Smad4 was performed using an automated immunostainer (Ventana Discovery XT autostainer, Ventana, USA). The antigens were retrieved by heat-induced antigen retrieval for 30 minutes with TRIS-EDTA buffer. The slides were stained with a polyclonal rabbit FSTL1 antibody (1:250; GeneTex, Taiwan), a polyclonal rabbit BMP4 antibody (1:300; GeneTex, Taiwan), and a polyclonal rabbit Smad4 antibody (1:500; Proteintech, USA). For phospho-Smad1/Smad5/Smad8 (p-Smad1/5/8), manual IHC staining was performed. Briefly, the slides were submitted to heat-induced antigen retrieval for 10 minutes with DAKO antigen retrieval buffer (pH 6) and incubated at 4 °C overnight with a polyclonal rabbit phospho-Smad1/Smad5/Smad8 antibody (1:50; Cell Signaling, USA) and then visualised using the 3, 3′-diaminobenzidine (DAB) peroxidase substrate kit (Vector Laboratories, USA).

### TMA immunohistochemistry interpretation

The IHC staining assessment was independently conducted by 2 pathologists who were blinded to patient outcome. For FSTL1 and BMP4, cytoplasmic IHC expressions of tumor cells in the cores were evaluated. For Smad4, nuclear and cytoplasmic immunoexpressions were both took into account. For p-Smad1/5/8, only nuclear IHC expression of tumor cells was scored. The immunoreactivity intensity and percentage were recorded. The intensity of staining was scored using a four-tier scale and defined as follows: 0, no staining; 1+, weak staining; 2+, moderate staining; 3+, strong staining. The extent of staining was scored by the percentage of positive cells: 0, 0–25%; 1+, 26–50%; 2+, 51–75%; 3+, 75–100%. The final IHC scores were obtained by multiplying the score of staining intensity and extent. All cases were divided into two groups according to the final IHC scores. Low IHC expression level was defined as a score less than 4 and a score more than and include 5 itself was defined as high expression.

### Public database

Public microarray database was used to validate the results from our cohort and to do further analysis. Survival analysis of large cohort microarray database with a total of 1324 NSCLC patients was performed using Kaplan-Meier plotter web resource (http://kmplot.com/analysis/) which includes The Cancer Genome Atlas (TCGA) cohort and 13 GSE datasets from Gene Expression Omnibus (GEO). The correlation between FSTL1 expression and molecular subtype of lung adenocarcinoma was evaluated through GSE31210 dataset.

### Chemicals and Antibodies

Recombinant FSTL1 protein was purchased from Sino Biological Inc. FSTL1 antibody was purchased from Proteintech.

### Cell lines and cell culture conditions

Lung cancer cell lines PC14 were maintained in DMEM supplemented with 10% FBS and 1% Penicillin-Streptomycin-Glutamine (PSG) (Invitrogen). PC14 was generated by Lee *et al*. at National Cancer Center Hospital, Tokyo, Japan^32^. Beas2B were acquired from the American Type Culture Collection.

### Animal studies

All animal work was done in accordance with a protocol approved by the Academia Sinica Institutional Animal Care and Use Committee (Protocol No: AS-IACUC-15-06-833). Female and male NOD-SCID mice (supplied by LASCO, Taiwan), age matched 6 to 8 weeks old, were used for tumor growth in a xenograft and a lung colonization metastasis model. For primary tumor growth assays, viable cells [5 × 10^6^ cells per 100 µl PBS] were injected subcutaneously into the back of mice. Primary tumor growth rates were analyzed by measuring tumor length (L) and width (W) and calculating volume through the formula LW^2^/2. After 6 weeks, mice were sacrified and tumor weight was measured.

### Statistical analysis

Statistics analysis was performed on SPSS 17.0 software (SPSS, Chicago, Illinois, USA). Estimates of the survival rates were calculated by the Kaplan-Meier method and compared by the log-rank test and univariate and multivariate analysis using Cox proportional hazards regression. The association between clinicopathological categorical variables and IHC expression were analyzed by chi-square test. Spearman correlation analysis was used to evaluate the correlation between IHC expressions of enrolled markers and Pearson correlation analysis was used to evaluate the correlation between RNA expressions of enrolled markers. For above analyses, a *P* value of <0.05 was considered statistically significant. Student’s t-test was used to compare RNA expressions between molecular subtypes. *P* values with the following levels were considered significant: *P < 0.05, **P < 0.01, ***P < 0.001.

## Results

### Low FSTL1, BMP4, and Smad4 expression are associated with poor prognosis in lung adenocarcinoma patients but not in squamous cell carcinoma patients

**Figure 1:**
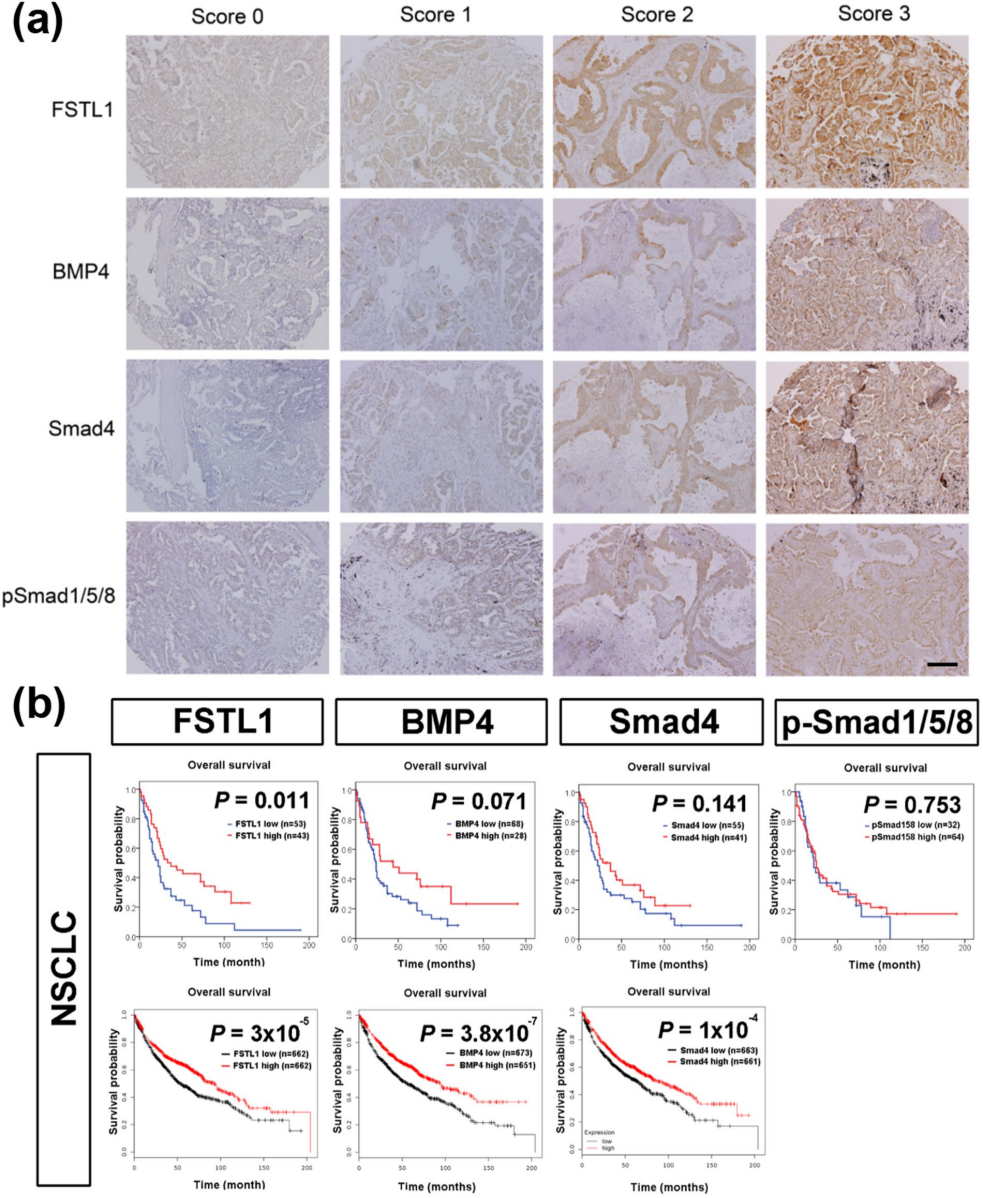
The prognostic significance of FSTL1, BMP4, Smad4, and p-Smad1/5/8 in non-small cell lung cancer. (**a**) IHC staining of FSTL1, BMP4, Smad4, and pSmad1/5/8. Photographs were taken at a magnification of 200X. Scale bars represent 100 μm. (**b**) Top panel: IHC expression study. Low FSTL1 IHC expression is significantly correlated with poor overall survival (*P* = 0.011). Patients with low BMP4 or Smad4 expression have a trend toward poor prognosis. No association is seen between p-Smad1/5/8 expression and survival. Lower panel: In survival analysis of large cohort microarray database using Kaplan-Meier plotter web resource, significant correlations are seen between low FSTL1, BMP4, and Smad4 RNA expression and poor overall survival. *P* value was obtained from Kaplan-Meier survival analysis.

To evaluate the importance of FSTL1-BMP4-Smad pathway in lung cancer, we first analyzed the prognostic value of FSTL1, BMP4, Smad4, and p-Smad1/5/8 (Fig. 1a). In our patient cohort, low FSTL1 IHC expression level was significantly correlated with poor overall survival (*P* = 0.011)(Fig. 1b, upper left). Patients with low BMP4 or low Smad4 IHC expression also had a trend toward poor prognosis (Fig. 1b, upper middle). No significant association between p-Smad1/5/8 IHC expression and prognosis was observed (Fig. 1b, upper right). The survival analysis results of public microarray database also showed significant association between low FSTL1 (*P* = 3 × 10^−5^), BMP4 (*P* = 3.8 × 10^−7^), and Smad4 (*P* = 1 × 10^−4^) RNA expression and poor overall survival (Fig. 1b, lower panel).

**Figure 2:**
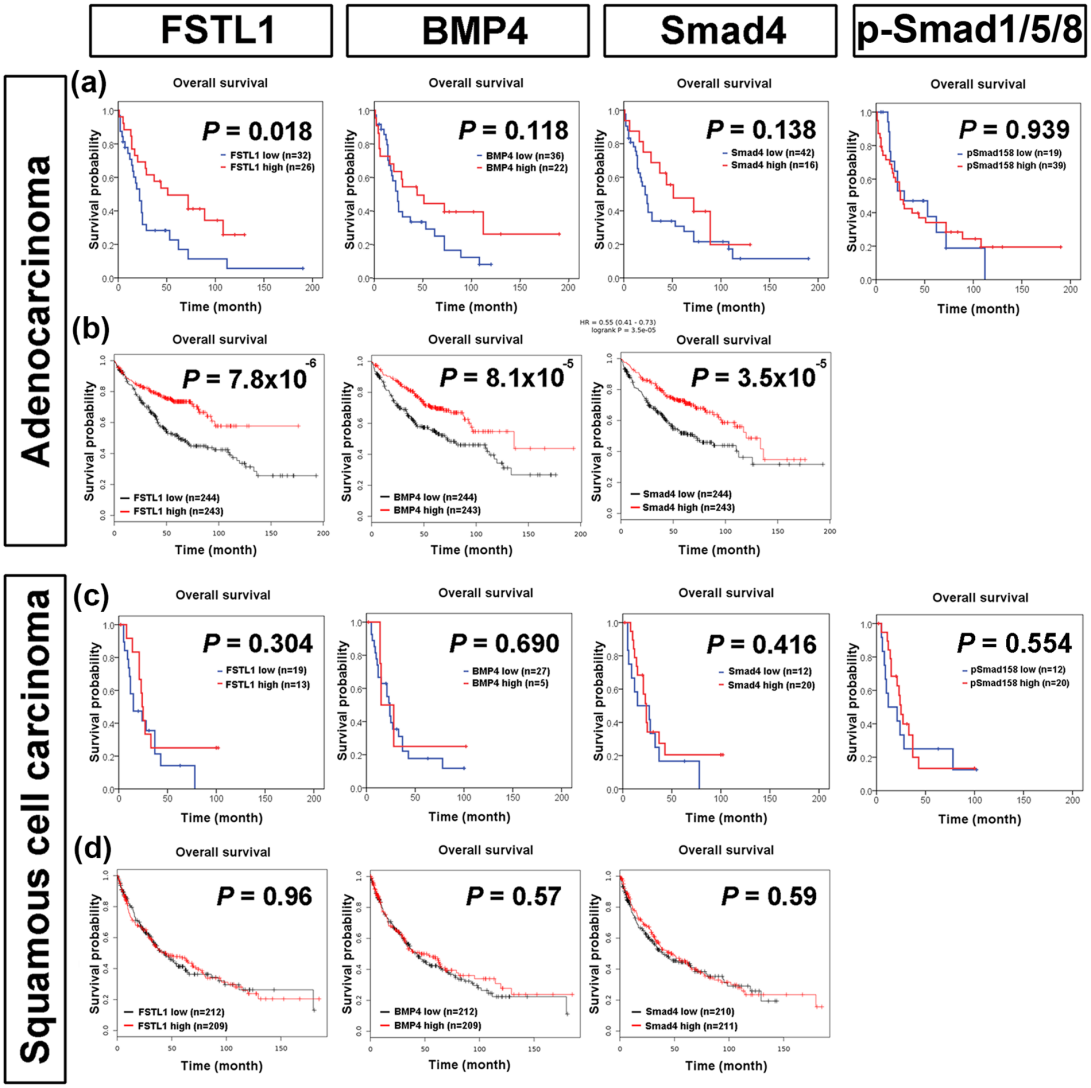
The prognostic significance of FSTL1, BMP4, Smad4, and p-Smad1/5/8 in lung adenocarcinoma and squamous cell carcinoma. In lung adenocarcinoma, (**a**) Kaplan-Meier analysis of overall survival of 58 lung adenocarcinoma patients stratified by FSTL1, BMP4, Smad4, and p-Smad1/5/8 protein levels. *P*-value was obtained from Kaplan-Meier survival analysis. (**b**) Kaplan-Meier analysis of overall survival of 487 lung adenocarcinoma patients stratified by FSTL1, BMP4, Smad4, and p-Smad1/5/8 mRNA levels from KM plotter web resource. *P*-value was obtained from Kaplan-Meier survival analysis. (**c**) Kaplan-Meier analysis of overall survival of 32 lung squamous cell carcinoma patients stratified by FSTL1, BMP4, Smad4, and p-Smad1/5/8 protein levels. *P*-value was obtained from Kaplan-Meier survival analysis. (**d**) Kaplan-Meier analysis of overall survival of 421 lung squamous cell carcinoma patients stratified by FSTL1, BMP4, Smad4, and p-Smad1/5/8 mRNA levels from KM plotter web resource. *P*-value was obtained from Kaplan-Meier survival analysis.

We further investigate the role of FSTL1-BMP4-Smad pathway in different histology subtypes of lung cancer. Low FSTL1 IHC expression retained its prognostic significance in lung adenocarcinoma (*P* = 0.018) (Fig. 2a). Patients with low BMP4 or low Smad4 IHC expression tend to have worse survival compared to those with high BMP4 or high Smad4. In contrast, no prognostic significance was seen in FSTL1, BMP4, and Smad4 IHC expression in squamous cell carcinoma (Fig. 2c). Consistent results were observed in the survival analysis results of public microarray database. There were significant correlations between low FSTL1 (*P* = 7.8 × 10^−6^), BMP4 (*P* = 8.1 × 10^−5^), and Smad4 (*P* = 3.5 × 10^−5^) RNA expression and poor overall survival (Fig. 2b) and no associations between FSTL1, BMP4, and Smad4 RNA expression and survival (Fig. 2d). For p-Smad1/5/8, there was no correlation between p-Smad1/5/8 IHC expression and prognosis in neither adenocarcinoma nor squamous cell carcinoma (Fig. 2a and c).

Univariate and multivariate analyses by the Cox proportional hazards model were also performed to confirm the prognostic significance of FSTL1-BMP4-pSmad158-Smad4 pathway in lung adenocarcinoma (Table 1). In univariate analysis, low FSTL1 expression, lymph node metastasis, and distant metastasis were all significantly correlated with unfavorable overall survival. In multivariate analysis, only low FSTL1 expression (hazard ratio [HR] = 2.55; 95% confidence interval [CI] = 1.04–6.24; *P* = 0.041) and lymph node metastasis (HR = 2.62; 95% CI = 1.26–5.46; *P* = 0.010) remained independently prognostic.

**Table 1:** Univariate and multivariate analysis of FSTL1 expression in lung adenocarcinoma patients.

Variables		Cox univariate analysis		Cox multivariate analysis	
		HR (95% CI)	*P*	HR (95% CI)	*P*
FSTL1	Low vs High	2.09 (1.11–3.94)	0.022	2.55 (1.04–6.24)	0.041
BMP4	Low vs High	1.66 (0.87–3.19)	0.126	1.22 (0.52–2.88)	0.643
p-Smad1/5/8	Low vs High	0.98 (0.50–1.89)	0.940	0.54 (0.24–1.21)	0.134
Smad4	Low vs High	1.70 (0.83–3.46)	0.148	1.30 (0.45–3.76)	0.632
T stage	T3–4 vs T1–2	1.78 (0.94–3.36)	0.077	1.09 (0.47–2.54)	0.843
N stage	N1–3 vs N0	2.52 (1.30–4.90)	0.006	2.62 (1.26–5.46)	0.010
M stage	M1 vs M0	3.01 (1.53–5.95)	0.001	1.98 (0.91–4.32)	0.087

HR: hazard ratio; CI: confidence interval.

### Significant correlations between FSTL1 expression and BMP4-Smad pathway in lung adenocarcinoma

**Figure 3:**
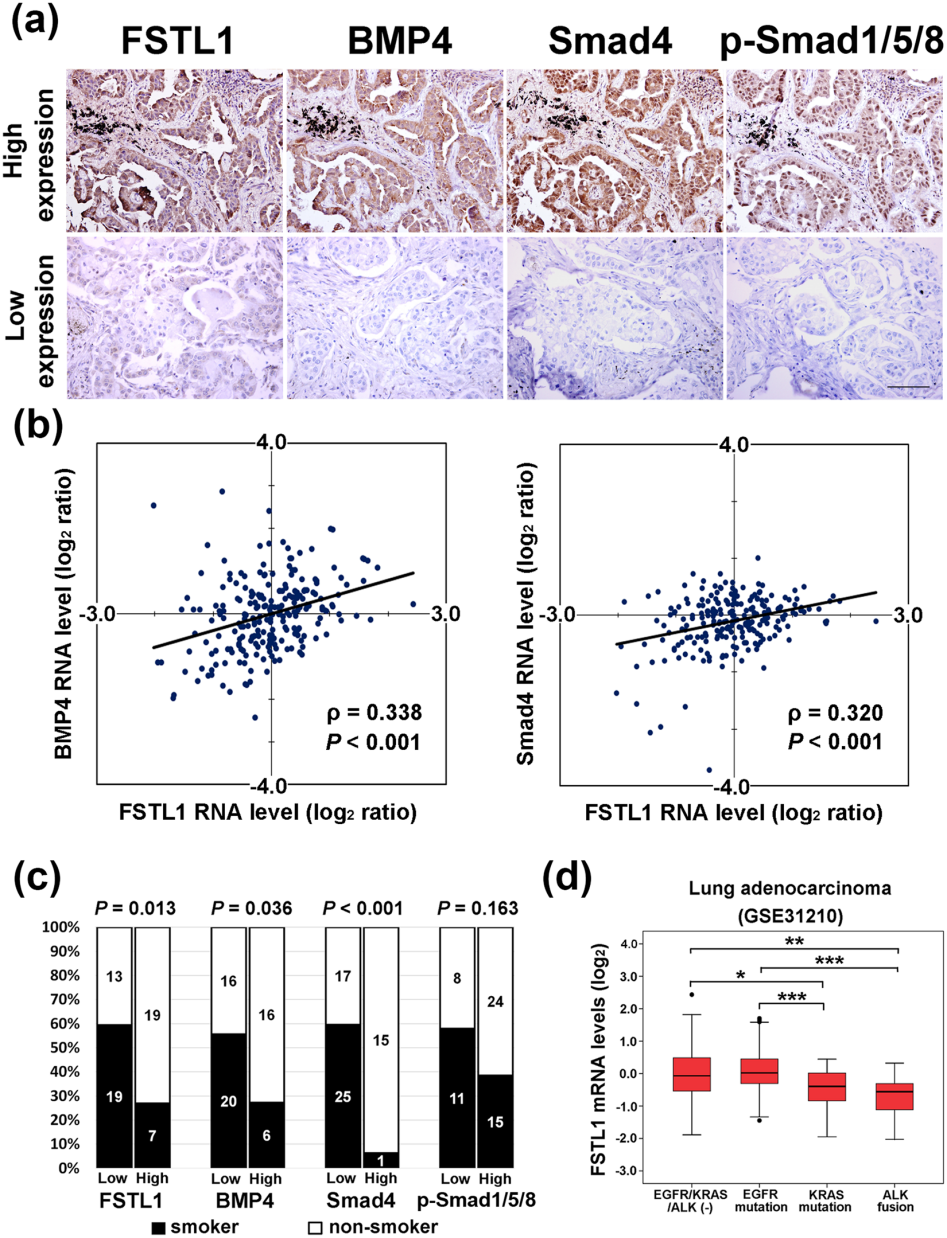
FSTL1 inversely correlates with BMP4, Smad, and p-Smad1/5/8 in LUAD. (**a**) Representative IHC staining pictures of lung adenocarcinoma showing one patient with high IHC expression of FSTL1, BMP4, Smad4, and p-Smad1/5/8 and the other patient with low IHC expression of FSTL1, BMP4, Smad4, and p-Smad1/5/8. Photographs were taken at a magnification of 200X. Scale bars represent 100 μm. (**b**) Left: Pearson correlation analysis between FSTL1 mRNA levels and BMP4 mRNA levels from GSE31210 microarray dataset. Right: Pearson correlation analysis between FSTL1 mRNA levels and Smad4 mRNA levels from GSE31210 microarray dataset. *P* value was obtained from Pearson correlation analysis. (**c**) Quantification of FSTL1, BMP4, Smad4, and p-Smad1/5/8 expression by immunohistochemistry analysis of lung cancer patients with or without smoking history. The number of patients for each group is shown in each column. *P*-value was obtained by Chi-square test. (**d**) FSTL1 mRNA expression profiles in patients with EGFR mutation, KRAS mutation, ALK fusion, or EGFR/KRAS/ALK triple-negative. *P < 0.05; **P < 0.01; ***P < 0.001.

After revealing the prognostic role of FSTL1, BMP4, and Smad4 in lung adenocarcinoma, we further analyzed their expression correlations. The results indicated that there were significant positive correlations among FSTL1, BMP4, Smad4, and p-Smad1/5/8 IHC expression in adenocarcinoma (Table 2). In contrast, there was either no correlation or weaker correlation in squamous cell carcinoma. Figure 3a showed representative IHC staining pictures of 2 cases of lung adenocarcinoma: one with high IHC expression of FSTL1, BMP4, Smad4, and p-Smad1/5/8 and the other with low IHC expression of FSTL1, BMP4, Smad4, and p-Smad1/5/8. We also analyzed these correlations in RNA level using public microarray dataset GSE31210, which includes 226 lung adenocarcinoma patients. Pearson correlation analysis showed FSTL1 mRNA level positively correlates with BMP4 mRNA level (ρ = 0.338, *P* < 0.001)(Fig. 3b, left). Significant positive correlation between FSTL1 mRNA level and Smad4 mRNA level was also observed (ρ = 0.320, *P* < 0.001) (Fig. 3b, right).

**Table 2:** The correlations between IHC expression level of FSTL1, BMP4, p-Smad1/5/8, and Smad4.

Spearman’s correlation	Adenocarcinoma				Squamous cell carcinoma			
	FSTL1	BMP4	p-Smad1/5/8	Smad4	FSTL1	BMP4	p-Smad1/5/8	Smad4
FSTL1	1				1			
BMP4	0.439 (0.001)	1			0.170 (0.353)	1		
p-Smad1/5/8	0.334 (0.010)	0.394 (0.002)	1		0.246 (0.174)	−0.022 (0.904)	1	
Smad4	0.530 (<0.001)	0.551 (<0.001)	0.266 (0.043)	1	0.378 (0.033)	0.156 (0.395)	0.333 (0.062)	1

### Low FSTL1 expression is associated with smoking history, ALK-fusion and KRAS mutation in lung adenocarcinoma

In clinicopathological analyses, low FSTL1 (*P* = 0.013), BMP4 (*P* = 0.036), and Smad4 IHC (*P* < 0.001) expression were positively correlated with smoking history (Fig. 3c). Patients with low FSTL1 expression also had a trend toward positive distant metastasis (*P* = 0.160) and higher pathological stage (*P* = 0.088) and low p-Smad1/5/8 expression was associated with higher T stage (*P* = 0.024) (Supplementary Table 1). In public dataset GSE31210, low FSTL1 RNA level was associated with lung adenocarcinoma patients with ALK-fusion or KRAS mutation compared to those with EGFR mutation or EGFR/KRAS/ALK triple-negative tumors (Fig. 3d). Similar trends were also shown in TCGA datasets (Supplementary Figure 1).

### FSTL1 down-regulation promotes the *in vivo* tumor growth in LUAD cells

**Figure 4:**
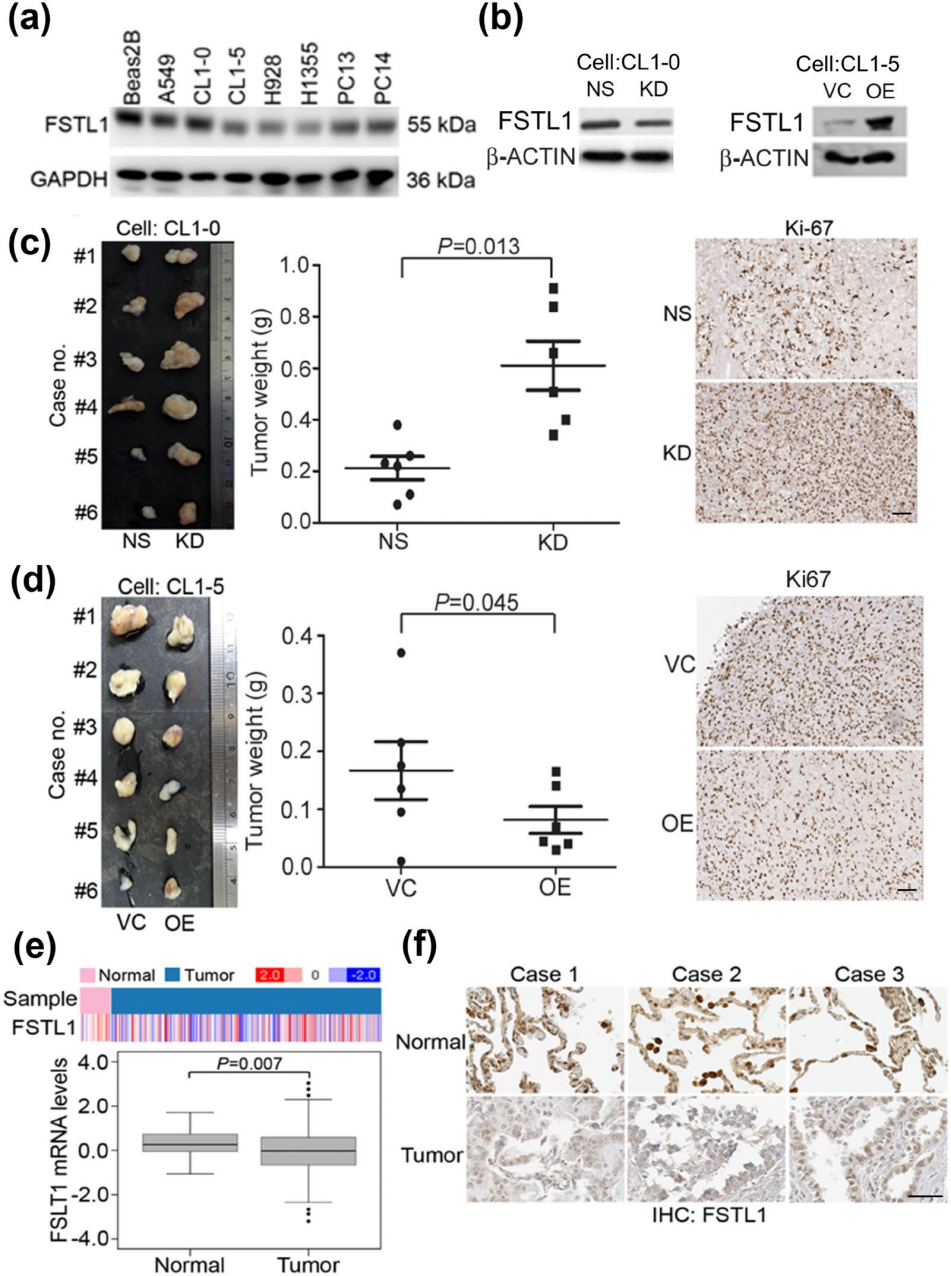
FSTL1 inhibits tumor growth in LUAD. (**a**) Endogenous FSTL1 protein expression in lung adenocarcinoma cell lines was analyzed by Western blot. GAPDH referred to the loading control. (**b**) Left: FSTL1 knockdown in CL1-0 cells were confirmed by Western blot analysis. Right: FSTL1 overexpression in CL1-5 cells were confirmed by Western blot analysis. β-actin referred to the loading control. NS: non-silencing shRNA control. KD: Knockdown using FSTL1 shRNA. Right: FSTL1 overexpression in CL1-5 cells was confirmed by Western blot analysis. VC: vector control; OE: FSTL1 overexpression. (**c**) Knockdown of FSTL1 expression promoted tumor growth *in vivo*. Left: Tumor images of mice injected with a non-silencing shRNA and FSTL1 shRNA-expressing CL1-0 cells. Middle: Tumor weight of individual mice 8 weeks after subcutaneous injection with CL1-0 cells infected with a non-silencing shRNA or FSTL1 shRNA. Right: Ki67 expression in mice tumor. Brown color represents Ki-67 IHC staining. Photographs were taken at a magnification of 100X. Scale bars represent 100 μm. (**d**) Left: Tumor images of mice injected with a vector control and FSTL1-expressing CL1-5 cells. Middle: Tumor weight of individual mice 6 weeks. Right: Ki67 expression in mice tumor. Brown color represents Ki-67 IHC staining. Photographs were taken at a magnification of 100X. Scale bars represent 100 μm. (**e**) Comparison of FSTL1 mRNA level in normal and tumor part in TCGA lung cancer patient database. (**f**) IHC staining of lung cancer patient. Photographs were taken at a magnification of 200X. Scale bars represent 100 μm. *P*-value was obtained from Student’s t-test analysis.

To determine the effect of FSTL1 perturbation on tumorigenesis, we first examined the endogenous FSTL1 protein expression in Beas2B normal lung epithelial cell and LUAD cell lines by Western blot analysis. We found FSTL1 protein expression levels in CL1-0 are higher than those in CL1-5 (Fig. 4a). Then we suppressed FSTL1 expression using FSTL1 specific shRNAs in CL1-0 cells (Fig. 4b, left). On the other hand, we ectopically overexpressed FSTL1 gene in CL1-5 cells (Fig. 4b; right). Subcutaneous tumorigenicity testing showed that knockdown of FSTL1 in CL1-0 cells promoted the tumor growth (Fig. 4c). On the other hand, FSTL1 overexpression in CL1-5 cells inhibited the tumor growth (Fig. 4d). Moreover, both mRNA (Fig. 4e) and protein (Fig. 4f) levels of FSTL1 were downregulated in tumors as compared to non-tumor tissues from patients with LUAD.

### FSTL1 inhibits nicotine-induced proliferation in lung cancer cells

**Figure 5:**
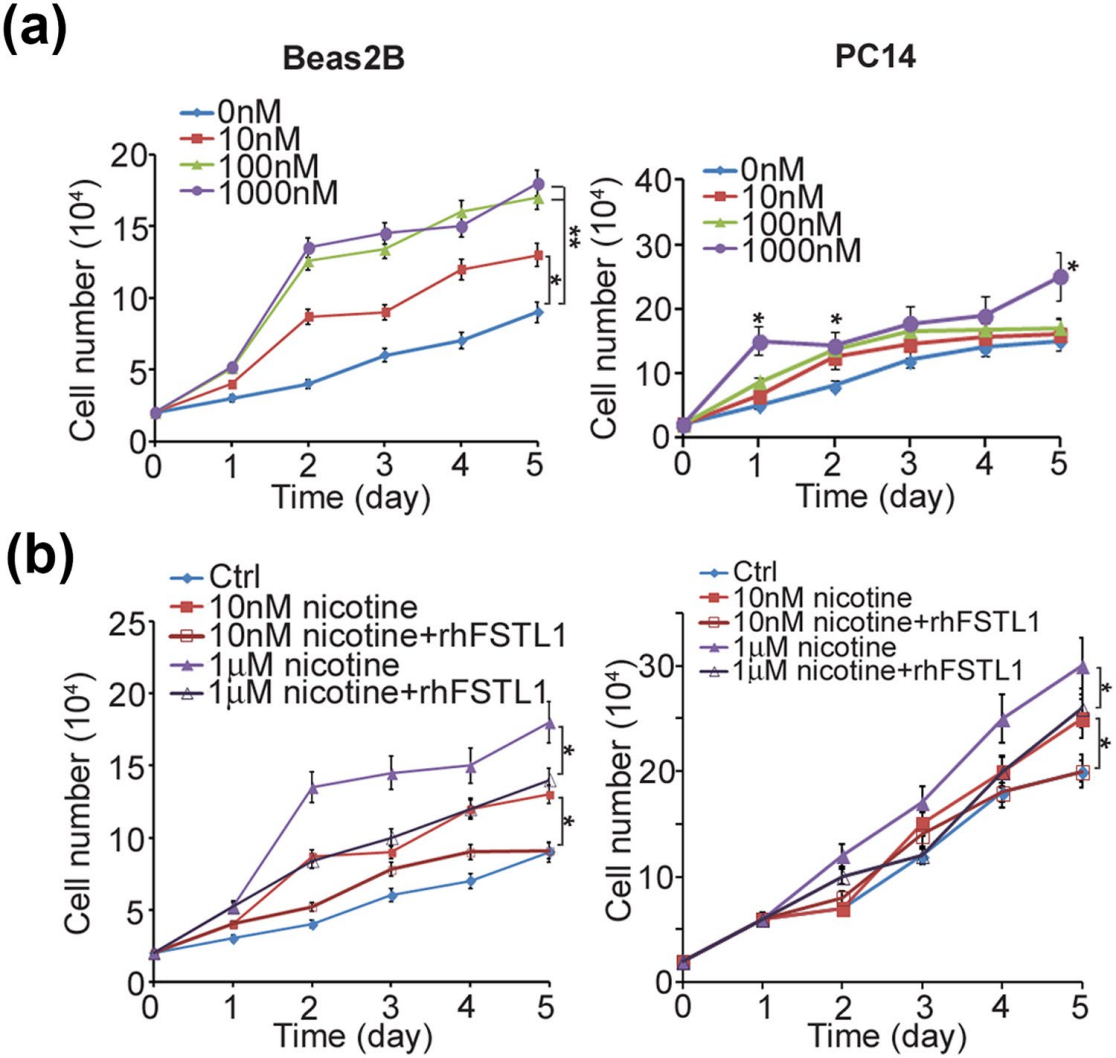
FSTL1 inhibits nicotine-induced proliferation in lung cancer cells. (**a**) Left: *In vitro* growth curve analysis of Beas2B upon nicotine treatment at indicated dose. Right: *In vitro* Growth curve analysis of PC14 upon nicotine treatment at indicated dose. Error bar: SD. *P < 0.05. **P < 0.01. (**b**) Left: *In vitro* growth curve analysis of Beas2B upon nicotine treatment at indicated dose with or without FSTL1 recombinant protein. Right: *in vitro* growth curve analysis of PC14 upon nicotine treatment at indicated dose with or without FSTL1 recombinant protein. Error bar: SD. *P < 0.05. *P*-value was obtained from Student’s t-test analysis.

Since low FSTL1 expression is associated with smoking history, we further investigated the effect of nicotine on lung cancer cells. Nicotine treatment significantly induced Beas2B and PC14 cells proliferation in a dose-dependent manner (Fig. 5a). Moreover, FSTL1 recombinant protein abolished nicotine-induced proliferation in multiple cell lines (Fig. 5b). Similar results were shown in lung adenocarcinoma cell line SK-LU-1 but not in small cell lung cancer cell line H2171 (Supplementary Figure 2). To identify the possible mechanism, we pre-treated chemical inhibitor of ERK/MAPK signaling pathway and then treated nicotine to verify the role of FSTL1. We found that extracellular FSTL1 protein levels were downregulated upon nicotine treatment in a time-dependent fashion (Supplementary Figure 3A). Moreover, p-ERK levels were increased by nicotine treatment and this induction can be prevented by PD98059 treatment (Supplementary Figure 3B). Meanwhile, extracellular FSTL1 protein levels were decreased by nicotine treatment and this downregulation was diminished by PD98059 (Supplementary Figure 3C). Furthermore, in cell proliferation assay, PD98059 treatment abolished nicotine-induced proliferation in a dose-dependent manner (Supplementary Figure 3D). Taken together, our results suggest nicotine treatment significantly downregulates extracellular FSTL1 levels. Nicotine-induced proliferation is mediated through ERK/MAPK signaling pathway and FSTL1 may attenuate nicotine-induced proliferation in lung cancer cells.

## Discussion

In the present study, we revealed that FSTL1-BMP4-Smad pathway plays an important role in cancer progression in lung adenocarcinoma but not in squamous cell carcinoma. The difference between the significance of FSTL1-BMP4-Smad pathway in adenocarcinoma and squamous cell carcinoma may be explained by its role in lung development. During lung organogenesis, FSTL1-BMP4-Smad pathway affects distal epithelial maturation through modulating surfactant expression which is secreted by type II pneumocytes^27, 29^, and alveolar epithelial cell hyperplasia with increased immature type II pneumocytes were observed in *Fstl1*-deficient mice^29^. Considering that type II pneumocytes are generally regarded as the origin of lung adenocarcinoma^33, 34^, it is reasonable that FSTL1-BMP4-Smad pathway dysregulation could result in strong prognostic impacts on lung adenocarcinoma patients. In contrast, the cell origin of squamous cell carcinoma is tracheal basal cells^33^. The mouse model showed FSTL1 deficiency affected proximal airway mainly in tracheal cartilage formation and had less influence on tracheal epithelium^27, 29^. Since FSTL1-BMP4-Smad pathway may have a more critical role in alveolar epithelium than in tracheal epithelium, different prognostic value of FSTL1-BMP4-Smad pathway in different lung cancer histologic types shown in our study reflects their different physiological function in different cell types.

The prognostic value of FSTL1 in lung adenocarcinoma highlighted in our study indicates the need to decipher the mechanism of FSTL1-regulated cancer progression. In lung adenocarcinoma of our patient cohort and public dataset, FSTL1 expression had positive correlation with BMP4-p-Smad1/5/8-Smad4 expression level and loss of FSTL1, BMP4, and Smad4 expression had a similar trend to predict poor prognosis. Since BMP4 has been shown to decrease proliferation and induce senescence of lung cancer cells via Smad-dependent pathway^17, 20^, FSTL1 may attenuate cancer progression through positively regulating BMP4-Smad pathway. However, on the contrary, FSTL1 is generally regarded as a BMP4 inhibitor under embryo development and physiological condition. During lung organogenesis, FSTL1 was reported to inhibit BMP4-modulated Smad signaling pathway and in turn decrease downstream pulmonary surfactant-associated protein C expression, something that is critical for alveolar maturation^27, 29, 35^. In ischemic heart disease, FSTL1 prevents myocytes apoptosis through inhibiting BMP4-p-Smad1/5/8 signaling and enhancing AMPK pathway^36^. Therefore, how FSTL1 modulates BMP4-Smad signaling pathway in lung adenocarcinoma should be clarified by further investigation.

Although loss of FSTL1 expression was shown to be a predictive marker of poor prognosis for lung adenocarcinoma in our study, it was reported to have contradictive roles in different kinds of tumor. FSTL1 expression inhibits cell growth and invasion of lung cancer cells, and the invasion ability could be blocked by FSTL1 antibody treatment^24^. In endometrial cancer and ovarian cancer, FSTL1 also has a tumor suppressive function to regulate cell proliferation, apoptosis, invasion and migration^37^. Downregulation of FSTL1 expression was also reported in various tumor cell lines including clear cell renal cell carcinoma^38^, colon cancer, and gastric cancer^22^. In contrast, a recent study focused on breast cancer showed that FSTL1 promotes bone metastasis through regulating tumor cell invasion ability and immune reprogramming. FSTL1 upregulation was also observed in glioblastoma and was correlated with poor prognosis in glioblastoma patients^39^. These research results again implicate that the function of FSTL1 is different in different cancer types.

Another interesting point worth to mention in our results is that lower FSTL1 expression was more commonly observed in patients with smoking habits. Although smoking is regarded to have more impact on the risk of squamous cell carcinoma and small cell carcinoma of lung than adenocarcinoma, adenocarcinoma patients with smoking history are the largest population in lung cancer patients^40, 41^. Cigarette smoking was reported to enhance lung cancer tumorigenicity and reduce apoptosis by attenuating TGF-β-mediated tumor suppression^42^. Here we showed the FSTL1 inhibition effect in nicotine-induced lung cancer cell proliferation. Since FSTL1 was primarily identified as a TGF-β inducible protein and BMP4 belongs to a ligand of TGF-β superfamily, if and how FSTL1-BMP4-Smad signaling is regulated by cigarette smoking is worth of future investigation. Besides, among molecular subtypes of lung adenocarcinoma, significant decrease of FSTL1 expression in KRAS mutation or ALK mutation tumor shown in our study also suggests that FSTL1 has variable influence on different molecular subtypes of lung adenocarcinoma and points out the direction of FSTL1 as a target for personalized therapeutic approach.

In conclusion, current study revealed that low FSTL1, BMP4, and Smad4 expression significantly predict poor prognosis in lung adenocarcinoma but not in squamous cell carcinoma. Same prognostic trends and positive correlation between the expression levels of FSTL1, BMP4, p-Smad1/5/8, and Smad4 indicates FSTL1 may regulate cancer progression through BMP4-p-Smad1/5/8-Smad4 signaling. Frequent decrease of FSTL1 expression in adenocarcinoma with KRAS mutation or ALK fusion and in smokers implies that FSTL1 has a critical role and may be a potential therapeutic target for some specific molecular types of lung adenocarcinoma.

## Electronic supplementary material

Supplementary information
