# Supplementary material for: Decrease of FSTL1-BMP4-Smad signaling predicts poor prognosis in lung adenocarcinoma but not in squamous cell carcinoma
Source: Sci Rep. 2017 Aug 29;7:9830. doi: 10.1038/s41598-017-10366-2 (PMC5575295; doi:10.1038/s41598-017-10366-2)

Decrease of FSTL1-BMP4-Smad signaling predicts poor prognosis in lung adenocarcinoma but not in squamous cell carcinoma

Jean Chiou[1,2], Chia-Yi Su[3], Yi-Hua Jan[2], Chih-Jen Yang[4], Ming-Shyan Huang[4], Yung-Luen Yu[1,5*], Michael Hsiao[2,5,6*]

[1]The PhD. Program for Cancer Biology and Drug Discovery, China Medical University, Taichung, Taiwan, [2]Genomics Research Center, Academia Sinica, Taipei, Taiwan, [3]Graduate Institute of Bioengineering, University of Washington, Seattle, WA, USA, [4]Department of Internal Medicine, Kaohsiung Medical University Hospital, Kaohsiung Medical University, Kaohsiung, Taiwan, [5]Graduate Institute of Cancer Biology and Center for Molecular Medicine, China Medical University, Taichung, Taiwan, [6]Department of Biochemistry, College of Medicine, Kaohsiung Medical University, Kaohsiung, Taiwan

*Correspondence to Dr. Michael Hsiao, Genomics Research Center, Academia Sinica, Taipei, Taiwan. Tel: +886-2-2787-1243; Fax: +886-2-2789-9931; E-mail: mhsiao@gate.sinica.edu.tw Or to Dr. Yung-Luen Yu, Graduate Institute of Cancer Biology and Center for Molecular Medicine, China Medical University, Taichung, Taiwan. Tel: +886-4-22053366; E-mail: ylyu@mail.cmu.edu.tw

Supplementary Table 1

Clinicopathological features	n	FSTL1 expression, n (%)		*P*	BMP4 expression, n (%)		*P*	Smad4 expression, n (%)		*P*	pSmad158 expression, n (%)		*P*
		Low (n=32)	High (n=26)		Low (n=36)	High (n=22)		Low (n=42)	High (n=16)		Low (n=36)	High (n=22)	
Age													
<65 y	35	19 (54.3)	16 (45.7)	0.867	24 (68.6)	11 (31.4)	0.208	27 (77.1)	8 (22.9)	0.320	12 (34.3)	23 (65.7)	0.760
≧65 y	23	13 (56.5)	10 (43.5)		12 (52.2)	11 (47.8)		15 (65.2)	8 (34.8)		7 (30.4)	16 (69.6)	
Gender													
Male	27	11 (40.7)	16 (59.3)	0.039	13 (48.1)	14 (51.9)	0.041	17 (63.0)	10 (37.0)	0.133	9 (33.3)	18 (66.7)	0.931
Female	31	21 (67.7)	10 (32.3)		23 (74.2)	8 (25.8)		25 (80.6)	6 (19.4)		10 (32.3)	21 (67.7)	
Smoking													
Non-smoker	32	13 (40.6)	19 (59.4)	0.013	16 (50.0)	16 (50.0)	0.036	17 (53.1)	15 (46.9)	<0.001	8 (25.0)	24 (75.0)	0.163
Smoker	26	19 (73.1)	7 (26.9)		20 (76.9)	6 (23.1)		25 (96.2)	1 (3.8)		11 (42.3)	15 (57.7)	
T status													
T1+T2	39	21 (53.8)	18 (46.2)	0.771	21 (53.8)	18 (46.2)	0.064	26 (66.7)	13 (33.3)	0.161	9 (23.1)	30 (76.9)	0.024
T3+T4	19	11 (57.9)	8 (42.1)		15 (78.9)	4 (21.1)		16 (84.2)	3 (15.8)		10 (52.6)	9 (47.4)	
N status													
N0	22	13 (59.1)	9 (40.9)	0.639	15 (68.2)	7 (31.8)	0.453	17 (77.3)	5 (22.7)	0.517	8 (36.4)	14 (63.6)	0.647
N1-N3	36	19 (52.8)	17 (47.2)		21 (58.3)	15 (41.7)		25 (69.4)	11 (30.6)		11 (30.6)	25 (69.4)	
M status													
M0	44	22 (50.0)	22 (50.0)	0.160	26 (59.1)	18 (40.9)	0.407	30 (68.2)	14 (31.8)	0.201	16 (36.4)	28 (63.6)	0.300
M1	14	10 (71.4)	4 (28.6)		10 (71.4)	4 (28.6)		12 (85.7)	2 (14.3)		3 (21.4)	11 (78.6)	
Pathological stage													
I + II	22	9 (40.9)	13 (59.1)	0.088	12 (54.5)	10 (45.5)	0.356	14 (63.6)	8 (36.4)	0.242	6 (27.3)	16 (72.7)	0.486
III + IV	36	23 (63.9)	13 (36.1)		24 (66.7)	12 (33.3)		28 (77.8)	8 (22.2)		13 (36.1)	23 (63.9)	
Recurrence													
No	35	19 (54.3)	16 (45.7)	0.867	21 (60.0)	14 (40.0)	0.689	25 (71.4)	10 (28.6)	0.836	10 (28.6)	25 (71.4)	0.402
Yes	23	13 (56.5)	10 (43.5)		15 (65.2)	8 (34.8)		17 (73.9)	6 (26.1)		9 (39.1)	14 (60.9)	

Supplementary Figure legends

Supplementary Figure 1. (A) In public TCGA dataset, significant correlations are seen between FSTL1 and BMP4 RNA level and between FSTL1 and Smad4 RNA level. *P* value was obtained from Pearson correlation analysis. (B) Lung adenocarcinoma with ALK-fusion or KRAS-mutation has more frequent low FSTL1 RNA expression compared to EGFR mutant or EGFR/KRAS/ALK-negative tumor. *P* value was obtained from Pearson correlation analysis.

Supplemetary Figure 2. FSTL1 recombinant protein inhibited nicotine-induced proliferation in SK-LU-1 but not in H2171 cells. (A) FSTL1 recombinant protein inhibited nicotine-induced proliferation in SK-LU-1 cells (lung adenocarcinoma cell line) proliferation with a dose-dependent manner. Error bar: SD. (B) H2171 cell line did not response to nicotine and FSTL1 treatment. Error bar: SD. *P* value was obtained from Student's t-test analysis.

Supplemetary Figure 3. FSTL1 inhibits nicotine-induced cell proliferation by ERK signal pathway. (A) Extracellular FSTL1 expression level were downregulated by nicotine (1μM) with a time-dependent manner. FSTL1 level was assayed by ELISA. Error bar: SD. *: $P<0.05$. **: $P<0.01$. *P* value was obtained from Student's t-test analysis. (B) PD98059 was pre-treated for 30 minutes before nicotine treatment (6hr). PD98059 prevented p-ERK induction after nicotine treatment. ERK referred to the loading controls. (C) PD98059 (50μM) inhibits nicotine-induced FSTL1 decreasing. PD98059 was pre-treated for 30 minutes before nicotine treatment (6hr). Extracellular FSTL1 expression level were assayed by ELISA. Error bar: SD. *: $P<0.05$. *P* value was obtained from Student's t-test analysis. (E) PD98059 inhibited nicotine-induced Beas2B (normal human bronchial epithelial cell) proliferation with

a dose dependent manner. Error bar: SD. *: $P<0.05$. **: $P<0.01$. *P* value was obtained from Student's t-test analysis.

Supplementary Figure 1

A

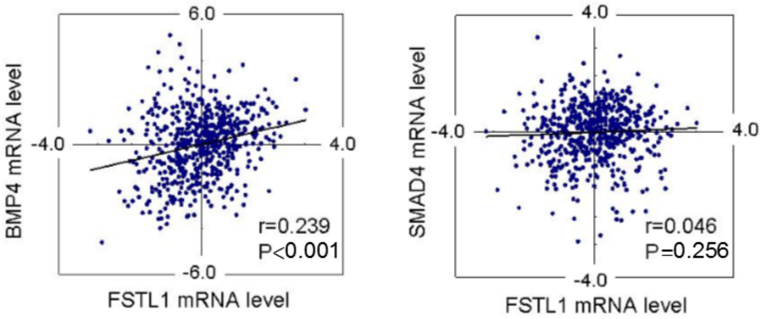

B

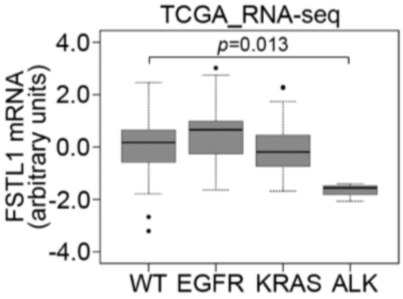

Supplementary Figure 2

A

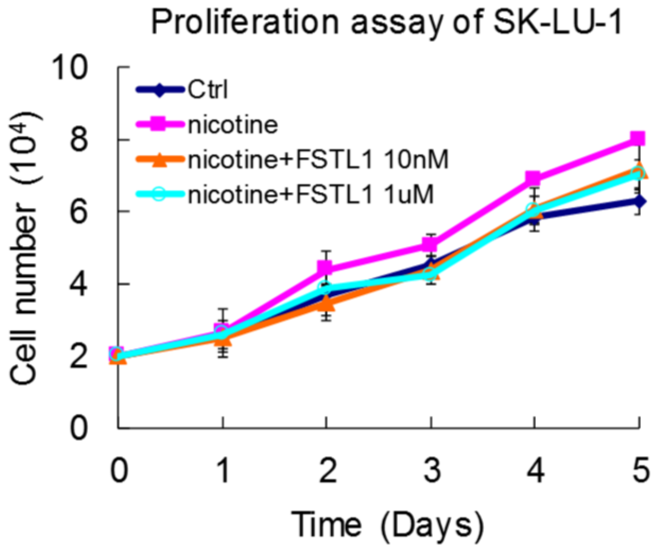

B

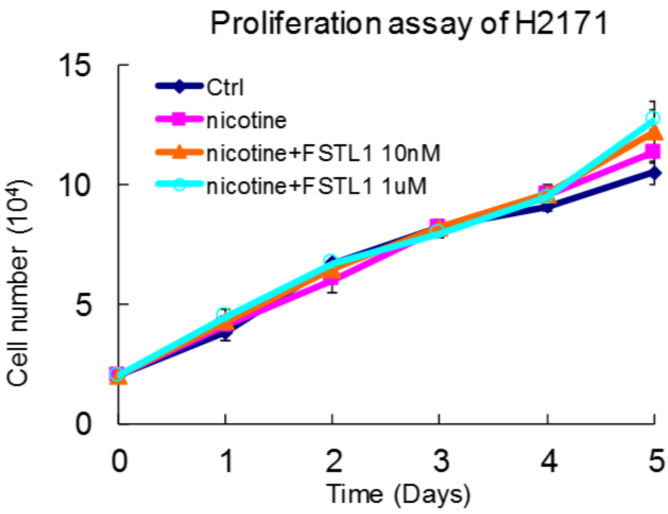

Supplementary Figure 3

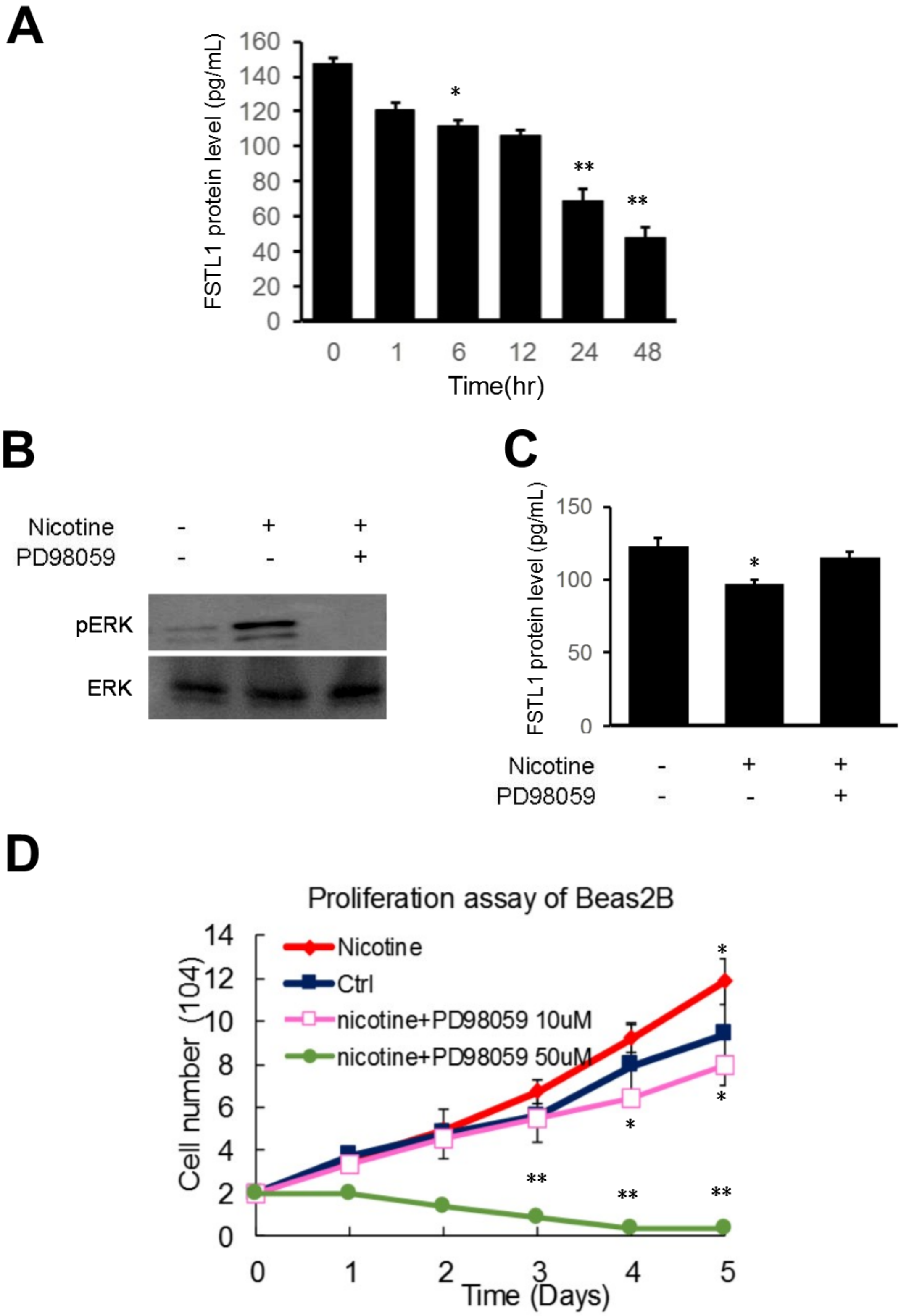

Original Raw Images Information

Figure 3(a): Full length Western blot images were taken by LAS-4000 Luminescent Image Analyzer
Figure 3(b) (left): Full length Western blot images were taken by X-ray films
Figure 3(b) (right): Full length Western blot images were taken by X-ray films.

Figure 3 (a)

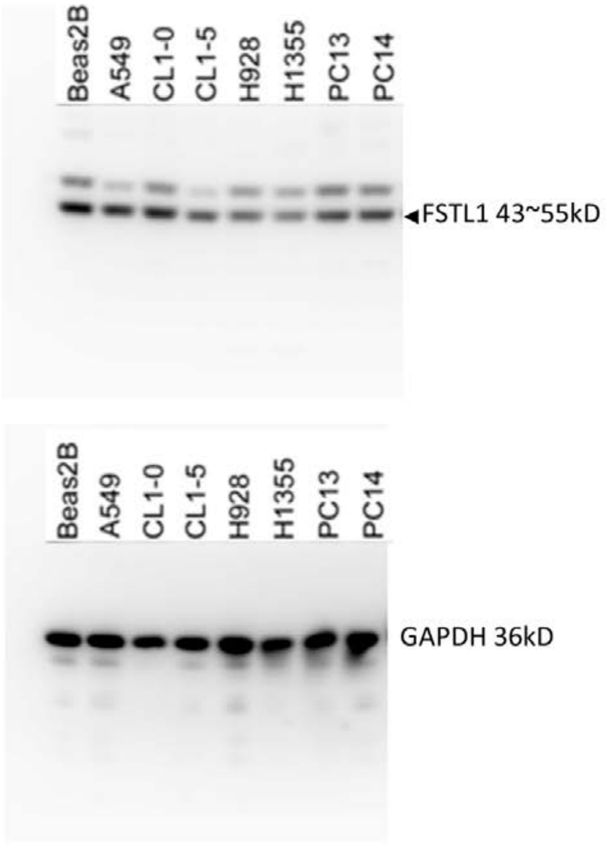

Figure 3 (b)

(Left)

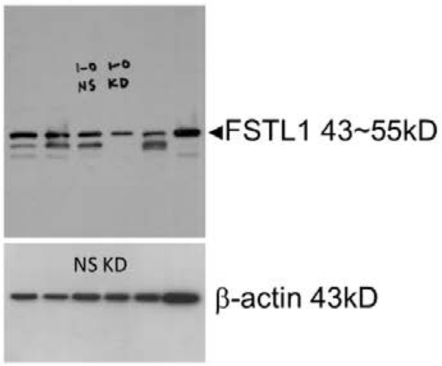

(Right)

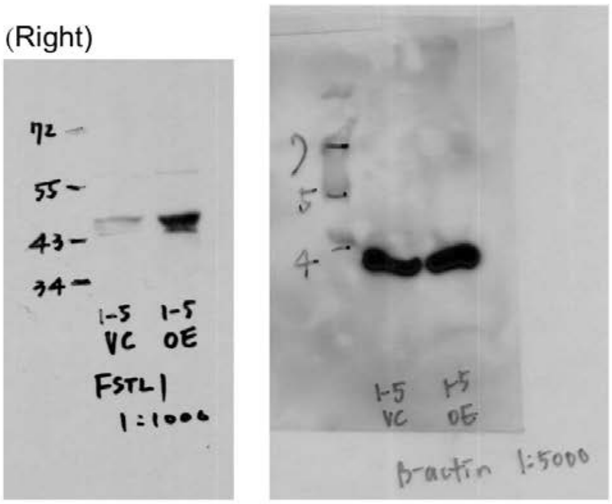

Supplement: Supplementary file 1 — Supplementary information [file 41598_2017_10366_MOESM1_ESM.pdf]
